# Early maladaptive schemas in episodic and chronic migraine in adolescents

**DOI:** 10.3389/fneur.2023.1128953

**Published:** 2023-04-21

**Authors:** Gülen Güler Aksu, Ozan Kayar, Ali Evren Tufan, Meryem Özlem Kütük, Ayşe Nur Özdağ Acarli, Damla Hazal Sucu, Bahar Taşdelen, Fevziye Toros, Aynur Özge

**Affiliations:** ^1^Department of Child and Adolescent Psychiatry, Mersin University School of Medicine, Mersin, Türkiye; ^2^Department of Psychology, Çankiri Karatekin University, Çankiri, Türkiye; ^3^Department of Child and Adolescent Psychiatry, Abant Izzet Baysal University, Bolu, Türkiye; ^4^Department of Child and Adolescent Psychiatry, Başkent University School of Medicine, Adana, Türkiye; ^5^Department of Neurology, Independent Physician, Istanbul, Türkiye; ^6^Department of Biostatistics and Medical Informatics, Mersin University School of Medicine, Mersin, Türkiye; ^7^Department of Neurology, Mersin University School of Medicine, Mersin, Türkiye

**Keywords:** adolescent, migraine, episodic, chronic, early maladaptive schemas, psychopathology

## Abstract

**Introduction:**

Psychotherapies, such as schema therapy, are receiving increasing attention in the management of pediatric headaches. The purpose of this study was to investigate early maladaptive schemas (EMSs) in adolescents with episodic migraine (EM) and chronic migraine (CM).

**Methods:**

This clinic-based, cross-sectional study consisted of 167 adolescents, aged 12–18, who were diagnosed with EM (*n* = 140) and CM (*n* = 27). The clinical characteristics of migraine, its accompanying symptoms, EMSs, the interrelationship of EMSs, depression, and anxiety were evaluated. We specifically analyzed psychopathology and abuse history as covariates in this study.

**Results:**

Defectiveness/shame, mistrust/abuse, abandonment/instability, enmeshment/undeveloped self, self-sacrifice, and subjugation schemas were more prevalent in the CM group. In terms of schema domains, the CM group scored significantly higher in disconnection/rejection and other orientations. Psychopathology did not affect the EMS scores, but a history of sexual abuse did. In patients with EM, a relationship was found between the variables of anxiety, depression, and five of the EMS domains. On the other hand, the CM group showed a significant relationship with anxiety, hypervigilance/inhibition, disconnection/rejection, and other orientation domains.

**Discussion:**

This study highlights the value of EMSs, anxiety, and depression in young people with EM and CM. Schema therapy and schema-based therapeutic interventions should be researched, especially in pediatric migraine, as they may potentially prevent the progression to treatment-resistant migraine.

## 1. Introduction

Episodic migraine (EM) and chronic migraine (CM) are two distinct clinical entities in terms of clinical, epidemiologic, sociodemographic, and comorbidity characteristics. Although CM is less common, it is far more debilitating and associated with a reduced quality of life (1), a higher rate of psychiatric and medical comorbidities (2), and an increased economic burden (3) than EM. EM progresses to CM at a rate of 2.5% per year (4). Age, gender, family history of headaches, increased headache frequency, acute medication overuse, obesity, caffeine, and psychopathologies are well-known risk factors for the development of CM (1–3). Psychiatric disorders have a close and complex relationship with headaches, based on a neurobiological and genomic background (5, 6). Anxiety, depression (5), cognitive structures (7, 8), personality traits (9), and coping styles (10) are some of the related psychological factors. Psychological stress and psychopathology may lead to the onset of headaches, the worsening of headache episodes, and the acceleration of headache chronicity (1, 3, 4, 11). However, there are still no clear data on how psychiatric disorders lead to worsening headache episodes or treatment-resistant and treatment-refractory migraine.

Beck emphasized maladaptive schemas, which are basic beliefs about the self, others, and the world (called cognitive schemas), that may lead to vulnerability to psychopathology (7). Young modified and updated Beck's cognitive model to identify different schemas (7, 12). In Young's model, early maladaptive schemas (EMSs) are significantly dysfunctional patterns of beliefs about oneself and one's interactions with others that develop during childhood and are elaborated throughout one's lifetime (12, 13). EMSs are present in all individuals with some degree of certain psychological flexibility but are more extreme and rigid in the presence of psychopathology (12–14). Childhood experiences such as trauma, neglect, abuse, and psychological stress are associated with the development of EMSs (12–14). The connection between EMSs and psychopathology in children and adolescents is less clear. The concept of EMS is still developing and may be fluid at this time (14). Thus, understanding the relationship between EMS, psychopathology, and migraine in the early years of life is crucial.

EMSs may be one of the overlooked underlying core factors in patients with headaches. Accordingly, a quick and accurate diagnosis of migraine in childhood and its contributing factors may help prevent the development of CM in adolescence and later in life (15). The study of EMSs in pediatric migraine seems to be essential. In this way, the progression to resistant migraine may be prevented by improving new and comprehensive treatment modalities, such as psychotherapies. Therefore, we aimed to investigate the following: the headache characteristics of youths with migraine, EMSs in youths with EM and CM, and the relationship between schema domains, anxiety, and depression scores in youths with EM and CM.

## 2. Materials and methods

### 2.1. Participants

This clinic-based, cross-sectional study was conducted as part of an ongoing Turkish Child and Adolescent Headache Database Project conducted by the Department of Child and Adolescent Psychiatry and Neurology at the Faculty of Medicine at Mersin University. This project included the data of patients with headaches who were admitted to the aforementioned Department of Child and Adolescent Psychiatry from 1 January 2004 to 1 April 2021. The inclusion criteria of the study were adolescents aged between 12 and 18 years of age, who were diagnosed with migraine according to the criteria of the International Classification of Headache Disorders, 3rd Edition (ICHD-3), (16) who presented to the center where the study was conducted between 1 November 2013 and 1 April 2021. The patients with organic mental disorders, psychotic disorders, intellectual disabilities, alcohol and substance abuse, symptoms of personality disorders, vision and hearing loss, a history of severe (with loss of consciousness) or recurrent head trauma in the past month, other headache types (e.g., tensiontype headache, secondary headache), with neurological and physical conditions, and the patients with missing data were excluded.

### 2.2. Instruments

#### 2.2.1. Sociodemographic information form

This form was developed by the researchers and included questions about the patient's age, gender, socioeconomic status, parental education level, family structure, marital status, parental psychiatric disorders, and history of abuse.

#### 2.2.2. Headache questionnaire form

The questionnaire was developed and partially validated by Ozge et al. to gather data on age, gender, education level, family history of headaches, and headache characteristics (17). The form was administered to the patients by a neurologist (AO), who also performed a thorough neurologic examination and a family interview, if necessary.

#### 2.2.3. Kovacs children's depression inventory (CDI)

The CDI was developed by Kovacs to evaluate depressive symptoms in children between the ages of 6 and 17. It consists of a 27-item scale, and each item is scored between 0 and 2. It has a cutoff score of 19 and a maximum score of 54 (18).

#### 2.2.4. The screen for child anxiety-related emotional disorders (SCARED)

SCARED is used to evaluate anxiety symptoms in children and consists of 41 items. It is a self-report on a 3-point Likert-type scale with scores ranging from 0 to 2 (0: not true or seldom true, 1: somewhat true or occasionally true, and 2: very true or frequently true) (19).

#### 2.2.5. Early maladaptive schema (EMS) questionnaire set for children and adolescents

**Figure 1:**
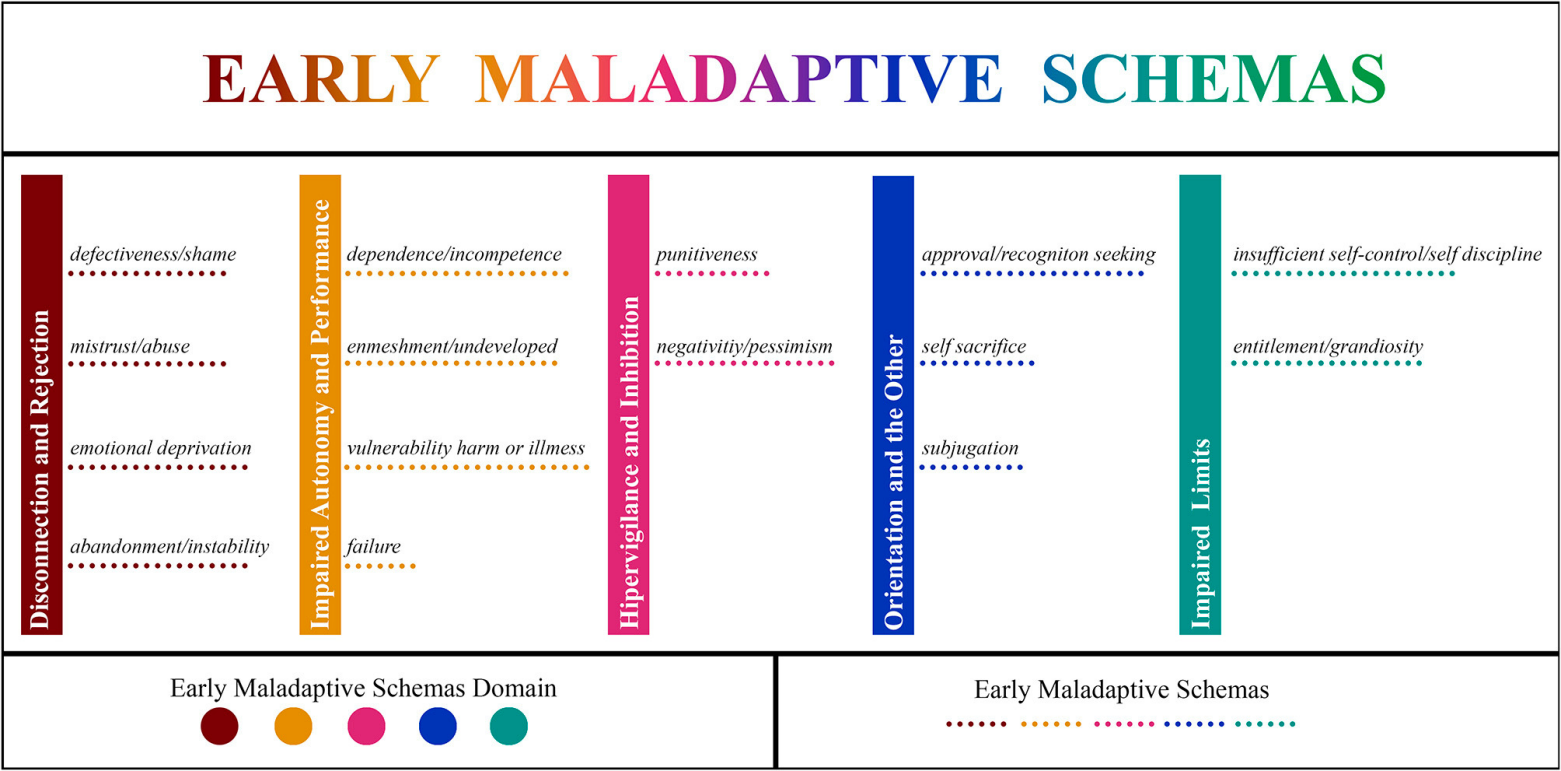
Illustration of 15 early maladaptive schemas and five schema domains consisting of disconnection and rejection, impaired autonomy and performance, hypervigilance and inhibition, impaired limits, and orientation to the others.

This scale was developed to evaluate EMSs in Turkish children between the ages of 10 and 16 (20). It is a self-report on a 5-point Likert scale with response options for each item ranging from 1 (completely disagree) to 5 (completely agree). The set consists of five schema domains, 97 items, and 15 EMSs. The 15 EMSs and the five schema domains are shown in Figure 1 (20).

### 2.3. Study procedure

A neurological examination was performed for each patient, and the ICHD-3 criteria (16) were used for the classification of headache types. Psychiatric diagnoses were assessed based on unstructured clinical interviews using the diagnostic criteria of the Diagnostic and Statistical Manual of Mental Disorders, Fifth Edition (DSM-5) (21). Adolescents were asked to complete all paper questionnaires independently in the waiting room of the outpatient clinic. All questionnaires were completed within 25–30 min. Sociodemographic characteristics, clinical characteristics, and accompanying symptoms of migraine and EMSs were analyzed. Furthermore, psychopathology and trauma history were also controlled in the subjects because they might influence the EMSs.

The study was conducted according to the principles of the Declaration of Helsinki and local laws and regulations. Institutional Research Board approval was obtained from the Faculty of Medicine at Mersin University (Number: E-78017789-050.01.04-1659432 Date: 26/05/2021).

### 2.4. Statistical analysis

The collected information was entered into a database prepared using SPSS version 23.0 (IBM Inc., Armonk, NY). The SPSS 23.0 and JAMOVI 2.0 software packages (the Jamovi project, 2021; https://www.jamovi.org) were used for analyses. Nominal variables were summarized as counts and frequencies, and quantitative data were summarized as either means and standard deviations or medians and interquartile ranges (IQRs), depending on the assumptions of normality. Bivariate comparisons of nominal variables were conducted using the chi-squared test with Yates' and Fisher's exact tests when appropriate. Quantitative variables were compared between groups using the Student's *t*-test for independent groups or the Mann-Whitney U-test. Schema domains and self-reported depression and anxiety symptoms between youths with EM and CM were compared using MANOVA followed by ANOVA. A history of sexual abuse and the presence of psychopathology were entered as covariates. Stepwise discriminant function analyses were conducted following MANOVA to evaluate the predictive functions of schema domains and self-reported depressive and anxiety symptoms for EM and CM. Relationships between schema domains and self-reported symptoms of depression and anxiety were evaluated using Pearson's correlation analyses. Due to the scarcity of studies on EMSs in adolescents with migraine and the exploratory nature of our study, we did not use correction for multiple comparisons.

Network analyses using EBICglasso were conducted to ascertain the relationships between schema domains and self-reported symptoms in patients with EM and CM. Significance was set at *p* = 0.05 (two-tailed). Effect sizes for significant findings were also reported.

## 3. Results

Out of 800 patients, only 456 patients completed the EMS scale. A total of 193 patients with other headache types, 15 patients with comorbidities, 32 patients with symptoms of personality disorder, five patients with head trauma, two patients with an intellectual disability, one patient with alcohol and substance abuse, and one patient with vision loss were excluded. A total of 40 patients were excluded due to missing data. A total of 167 patients (*n* = 112, 67.1% female patients) diagnosed as suffering from EM (*n* = 140, 87.0%) and CM (*n* = 27, 23.0%) met the study inclusion criteria. The groups were similar in terms of female preponderance (66.4% episodic vs. 70.4% chronic, χ^2^ = 0.03, *p* = 0.861, Yates'). Adolescents with EM and CM did not differ significantly in terms of their own and their parents' sociodemographic features. The sociodemographic characteristics of the respondents and their families according to migraine type are illustrated in Table 1.

**Table 1:** Sociodemographic features of the respondents and their families according to migraine type.

**Mean (SD) or** ***n*** **(%)**		**EM (*n* = 140)**	**CM (*n* = 27)**	t/ p^*^or χ^2^/p^**^
Age (years)		15.4 (1.9)	15.4 (1.9)	0.591/ 0.555
Maternal age (years)		41.8 (6.1)	42.6 (6.4)	−0.252/ 0.801
Paternal age (years)		46.3 (7.2)	47.2 (6.1)	−0.379/ 0.706
Maternal education	Primary	69 (49.3)	12 (52.2)	2.7/ 0.440
	High school	44 (31.4)	4 (17.4)	
	College	21 (15.0)	5 (21.7)	
	University or higher	6 (4.3)	2 (8.7)	
Paternal education	Primary	72 (51.4)	12 (54.5)	4.0/ 0.259
	High school	39 (27.9)	3 (13.6)	
	College	19 (13.6)	6 (27.3)	
	University or higher	10 (7.1)	1 (4.5)	
Maternal psychopathology		31 (22.1)	3 (11.1)	1.7 / 0.296
Paternal psychopathology		18 (12.9)	6 (22.2)	0.9/ 0.332
SES	Low	36 (25.7)	6 (22.2)	3.4/ 0.184
	Middle	83 (59.3)	13 (48.1)	
	High	21 (15.0)	8 (29.6)	
BMI (kg/m^2^)		21.8 (4.4)	22.1 (4.7)	−0.617/ 0.538

EM, episodic migraine; CM, chronic migraine; BMI, body mass index; SD, standard deviation; SES, socioeconomic status.

^*^t-test for independent samples. ^**^Chi-square test.

More than half of the youths in both groups with EM (*n* = 72, 51.4%) and CM (*n* = 16, 59.3%) met the criteria for psychopathology, with no significant difference between the groups (χ^2^ = 0.29, *p* = 0.592, Yates'). The diagnoses of adolescents with migraine according to temporal features are illustrated in Table 2.

**Table 2:** Psychiatric diagnoses of the respondents according to migraine type.

***n* (%)**	**EM (*n* = 140)**	**CM (*n* = 27)**
Attention-deficit/hyperactivity disorder	26 (18.6)	7 (25.9)
Oppositional defiant disorder	1 (0.7)	0 (0.0)
Learning disorders	3 (2.1)	0 (0.0)
Obsessive compulsive disorder	6 (4.3)	3 (11.1)
Generalized anxiety disorder	31 (22.1)	4 (14.8)
Social anxiety disorder	9 (6.4)	0 (0.0)
Separation anxiety disorder	2 (1.4)	0 (0.0)
Post-traumatic stress disorder	3 (2.1)	0 (0.0)
Major depressive disorder	13 (9.3)	2 (7.4)
Disruptive mood dysregulation disorder	1 (0.7)	0 (0.0)
Bipolar disorder	1 (0.7)	0 (0.0)
Panic disorder	6 (4.3)	0 (0.0)
Tic disorder	1 (0.7)	1 (3.7)

EM, episodic migraine; CM, chronic migraine.

For both EM and CM, the median number of diagnoses was 1.0 (IQR = 1.0) with no significant difference between the groups (Mann–Whitney *U*-test, Z = −0.4, *p* = 0.697). A history of sexual abuse was reported by five individuals with EM (3.6%); there were no reports of sexual abuse among youths with CM.

Features of pain in youths with EM and CM are illustrated in Table 3.

**Table 3:** Pain features in adolescents with migraine according to type.

**Median (IQR)**	**EM (*n* = 140)**	**CM (*n* = 27)**	Z/p^*^
Age at onset (years)	13.0 (3.0)	13.0 (4.0)	−0.7/0.508
Pain duration (months)	24.0 (27.0)	24.0 (30.0)	−0.6/0.558
Frequency/month	10.0 (6.8)	25.0 (14.0)	–***7.5***/*** <0.001***
Episode duration (minutes)	120.0 (210.0)	240.0 (675.0)	−1.4/0.178
Pain severity in the episode (VAS)	8.0 (1.8)	7.0 (2.0)	−0.2/0.884

IQR, interquartile range; EM, episodic migraine; CM, chronic migraine; VAS, visual analog scale (0–10). ^*^Mann–Whitney *U*-test. Bold italic values mean *p* < 0.05.

In bivariate comparisons, except for the frequency of migraine episodes/month, other pain features did not differ significantly between the groups. Symptoms associated with migraine episodes are illustrated in Table 4.

**Table 4:** Symptoms of migraine episodes according to type.

**N (%)**		**EM (*n* = 140)**	**CM (*n* = 27)**	χ^2^/p^*^
Nausea and/or vomiting		79 (56.4)	16 (59.3)	0.0/0.952
Photophobia and/or phonophobia		131 (93.6)	(92.6)	0.0/0.693
Osmophobia		66 (47.1)	15 (55.6)	0.4/0.555
Vertigo		92 (65.7)	20 (74.1)	0.4/0.534
Dizziness		107 (76.4)	21 (77.8)	0.0/0.999
Cranial autonomic features	Lacrimation	56 (40.0)	17 (63.0)	***4.0***/***0.047***
	Blurred vision	35 (25.0)	9 (33.3)	0.4/0.508
	Bloodshot eyes	59 (42.1)	11 (40.7)	0.0/0.999
	Photopsia	52 (37.1)	17 (63.0)	***5.2***/***0.023***
	Flushing	60 (42.9)	9 (33.3)	0.5/0.480
	Rhinorrhea	34 (24.3)	4 (14.8)	1.2/0.329
	Nasal stuffiness	43 (30.7)	9 (33.3)	0.0/0.966
Pain character	Throbbing	132 (94.3)	21 (77.8)	***6.0***/***0.014***
	Piercing	80 (57.1)	13 (48.1)	0.4/0.516
	Pressing	91 (65.0)	16 (59.3)	0.1/0.726
	Heaviness	62 (44.3)	11 (40.7)	0.0/0.898
Relieving factors	Resting in the dark	124 (88.6)	19 (70.4)	***4.7***/***0.030***
	Eating	13 (9.3)	4 (14.8)	0.8/0.483
	NSAIDs	113 (80.7)	21 (77.8)	0.0/0.931
	Vomiting	19 (13.6)	3 (11.1)	0.1/0.999
	Massage	82 (58.6)	13 (48.1)	0.6/0.430
	Cold application	13 (9.3)	0 (0.0)	–/–
	Cranial pressure	17 (12.2)	0 (0.0)	–/–

EM, episodic migraine; CM, chronic migraine; NSAIDs, non-steroidal anti-inflammatory drugs. ^*^Chi-square test (with Yates' and Fisher's tests as needed). Bold italic values mean *p* < 0.05.

When the pain characteristics were compared between groups, some autonomic symptoms, such as lacrimation and photopsia, were found to be significantly more frequent in respondents with CM (with small effect sizes, Phi 0.17 and 0.19, respectively). A throbbing pain was reported significantly more frequently in youths with EM (Phi = 0.22). Youths with EM also reported relief from resting in a dark room significantly more frequently than those with CM (Phi = 0.19). Among the adolescents with EM, more than half (*n* = 92, 55.1%) reported no auras, and the remaining youths experienced auras.

EMSs and schema domains were compared between youths with EM and CM using MANOVA. The covariance matrices were homogenous (Box's M, χ^2^[28] = 28.3, *p* = 0.451), and Levene's test showed that the error variances for the emotional deprivation, negativity/pessimism schema, and hypervigilance domains were not equal (*p* = 0.021, *p* = 0.002, and *p* = 0.004, respectively). Therefore, Pillai's trace was used for comparison. Adolescents with EM and CM differed significantly in terms of EMSs and schema domains (F [15.0, 151.0] = 1.9, *p* = 0.028, partial η^2^ = 0.16). Follow-up univariate ANOVAs are presented in Table 5.

**Table 5:** Comparison of adolescents with episodic (EM) and chronic migraine (CM) in terms of early maladaptive schemas and schema domains.

	**EM (*n* = 140)**	**CM (*n* = 27)**	F/p^*^	**Partial η^2^**
Defectiveness/shame	12.7 (4.4)	14.8 (3.7)	**5.7**/**0.018**	**0.03**
Mistrust/abuse	17.6 (6.7)	21.6 (5.9)	**8.5**/**0.004**	**0.05**
Emotional deprivation	23.5 (10.0)	27.1 (7.8)	3.2/0.078	0.02
Abandonment/instability	9.3 (3.7)	10.9 (3.7)	**4.2**/**0.043**	**0.03**
Failure	18.3 (7.0)	20.6 (6.3)	2.5/0.117	0.02
Dependence/incompetence	15.8 (5.9)	17.3 (5.5)	1.6/0.214	0.01
Enmeshment/undeveloped self	8.0 (3.2)	9.7 (3.8)	**6.6**/**0.011**	**0.04**
Vulnerability to harm/illness	9.7 (3.6)	10.2 (3.0)	0.5/0.491	0.00
Entitlement/grandiosity	42.1 (12.4)	41.1 (9.6)	0.2/0.685	0.00
Insufficient self-control/self-discipline	15.1 (4.9)	14.9 (4.0)	0.1/0.787	0.00
Self-sacrifice	10.2 (4.4)	13.0 (3.9)	**9.9**/**0.002**	**0.06**
Subjugation	16.0 (5.4)	18.4 (6.3)	**4.3**/**0.039**	**0.03**
Approval/recognition seeking	10.5 (3.8)	11.4 (3.7)	1.2/0.273	0.01
Negativity/Pessimism	25.7 (8.7)	28.8 (6.0)	3.3/0.073	0.02
Punitiveness	13.9 (4.2)	14.9 (3.3)	1.3/ 0.265	0.01
Disconnection/rejection domain	63.1 (21.5)	74.5 (17.3)	**6.7**/**0.010**	**0.04**
Impaired autonomy domain	65.9 (16.5)	68.1 (14.4)	0.5/0.505	0.00
Impaired Limits domain	15.1 (4.9)	14.9 (4.0)	0.1/0.787	0.00
Other orientation domain	36.7 (10.9)	42.8 (11.1)	**7.2**/**0.008**	**0.04**
Hypervigilance domain	39.6 (11.5)	43.8 (8.0)	3.4/0.066	0.02

EM, episodic migraine; CM, chronic migraine.

^*^Univariate ANOVAs (Bonferroni corrected). Bold values mean *p* < 0.05.

**Figure 2:**
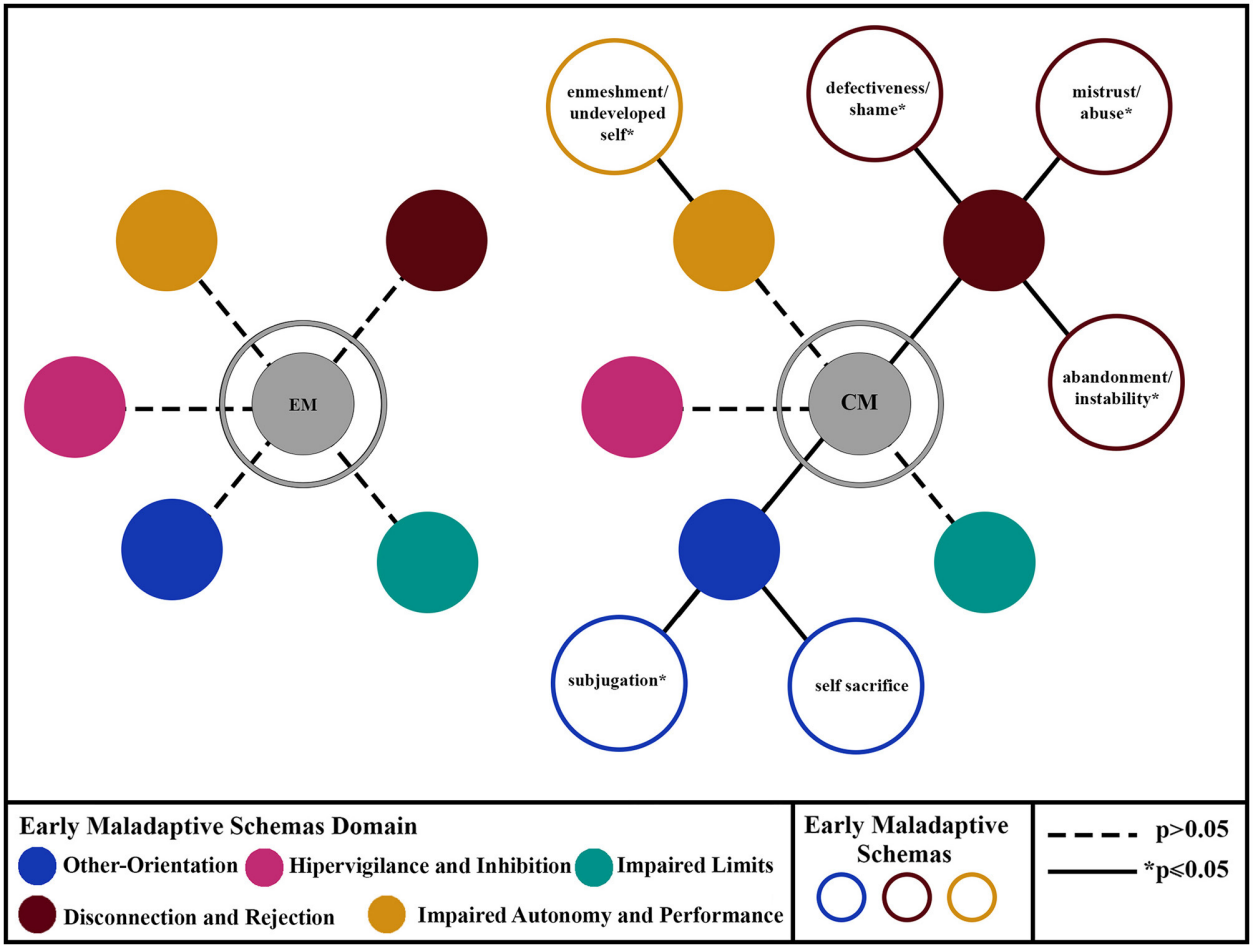
Illustration showing a summary of the EMSs and EMS domain scores from bivariate comparisons between EM and CM.

In bivariate comparisons, youths with CM scored significantly higher on defectiveness/shame, mistrust/abuse, abandonment/instability, enmeshment/undeveloped self, self-sacrifice, and subjugation schemas. As for the schema domains, adolescents with CM scored significantly higher on disconnection/rejection and other orientation domains (*p* = 0.01 and *p* = 0.008, respectively) (Figure 2).

The inclusion of sexual abuse history and the presence of psychopathology did not affect the difference between the groups in terms of EMS scores (F [15.0, 149.0] = 1.9, *p* = 0.027, partial η^2^ = 0.16, Pillai's Trace). The presence of psychopathology did not affect EMS scores (F [15.0, 149.0]=1.2, *p* = 0.313, partial η^2^ = 0.10), but sexual abuse history did (F [15.0, 149.0] = 1.8, *p* = 0.038, partial η^2^ = 0.15, both Pillai's Trace). Youths with a history of sexual abuse had significantly higher scores on the defectiveness/shame, mistrust/abuse, emotional deprivation, dependence/incompetence, vulnerability to harm/illness, entitlement/grandiosity, self-sacrifice, and negativity/pessimism subscales. They also scored significantly higher on the disconnection/rejection, impaired autonomy, and hypervigilance domains.

Stepwise discriminant function analysis with EMSs revealed just one function involving the other orientation domain (Wilks's λ= 0.96, χ^2^ = 7.0, *p* = 0.008). The correlations of the disconnection/rejection (*r* = 0.73), hypervigilance (*r* = 0.59), impaired autonomy (*r* = 0.51), and impaired limit (*r* = 0.30) domains with the discriminant function were lower. This function was able to correctly classify 99.3% of adolescents with EM and 96.3% of those with CM, for an overall accuracy of 83.8%. When SCARED and CDI scores were entered into a stepwise discriminant function analysis, only one function including SCARED scores emerged (Wilks's λ= 0.94, χ^2^ = 7.5, *p* = 0.006). This function correctly classified 100.0% of youth with EM and none of those with CM, for an overall accuracy of 83.8%.

The mean CDI scores for youths with EM and CM were 15.9 (SD = 8.8) and 21.3 (SD = 9.8), respectively. The mean SCARED scores for adolescents with EM and CM were 31.9 (SD = 15.6) and 42.3 (SD = 13.4), respectively. The covariance matrices (Box's M, F [3.0, 14492.9] = 1.1, *p* = 0.363) and error variances (*p* = 0.589 and *p* = 0.375, respectively, Levene's test) were equal. Youths with EM and CM differed significantly in terms of depressive and anxiety symptoms (F [2.0, 131.0] = 4.7, *p* = 0.011, partial η^2^ = 0.07, Wilks's λ) with scores for CM being greater. The inclusion of sexual abuse and psychopathology as covariates did not affect the difference between EM and CM (F [2.0, 129.0] = 5.2, *p* = 0.007, partial η^2^ = 0.08, Wilks's λ). The presence of sexual abuse did not affect the self-reported depressive and anxiety symptoms (F [2.0, 129.0] = 0.3, *p* = 0.769, partial η^2^ = 0.00), but youths with a psychiatric diagnosis had significantly higher scores (F [2.0, 129.0] = 20.5, *p* < 0.001, partial η^2^ = 0.24, both Wilks's λ).

In adolescents with EM, age correlated significantly with the impaired limits domain of the EMSs (*r* = 0.24, *p* = 0.004). CDI and SCARED scores correlated positively with disconnection/rejection (*r* = 0.54, *p* < 0.001 and *r* = 0.45, *p* < 0.001, respectively), impaired autonomy (*r* = 0.38, *p* < 0.001 and *r* = 0.27, *p* = 0.003, respectively), impaired limits (*r* = 0.41, *p* < 0.001 and *r* = 0.23, *p* = 0.015, respectively), other orientation (*r* = 0.44, *p* < 0.001 and *r* = 0.29, *p* = 0.002, respectively), and hypervigilance (*r* = 0.60, *p* < 0.0001 and *r* = 0.53, *p* < 0.001, respectively) domains. When age was controlled, these correlations did not change significantly.

In adolescents with CM, age did not correlate with EMS domains. SCARED scores correlated significantly and positively only with the disconnection/rejection (*r* = 0.59, *p* = 0.006), and other orientations (*r* = 0.45, *p* = 0.045) domains. There were no significant correlations between CDI scores and EMS domains. When age was controlled, these correlations did not change significantly.

Network analyses with EBICglasso revealed a network of seven nodes with 15/21 non-zero edges for EM (sparsity = 0.29) and seven nodes with 3/21 non-zero edges (sparsity = 0.86) for CM. Centrality and clustering measures per variable for the EM and CM networks are illustrated in Table 6.

**Table 6:** Measures of centrality and clustering (Barrat) per variable for adolescents with episodic (EM) and chronic migraine (CM).

**Variable**	**EM (*****n*** = **140)**					**CM (*****n*** = **27)**				
	**Betweenness**	**Closeness**	**Strength**	**Expected influence**	**Clustering**	**Betweenness**	**Closeness**	**Strength**	**Expected influence**	**Clustering**
Disconnection/ rejection	0.57	1.03	1.41	1.41	0.47	2.27	0.00	2.00	2.00	0.00
Impaired autonomy	−0.23	−0.75	−0.25	−0.25	−0.87	−0.38	0.00	−0.80	−0.80	0.00
Impaired limits	−0.23	−1.15	−1.06	−1.06	−0.96	−0.38	0.00	−0.80	−0.80	0.00
Other orientation	−0.23	0.50	−0.23	−0.23	0.97	−0.38	0.00	0.13	0.13	0.00
Hypervigilance	1.37	1.25	1.26	1.26	−0.39	−0.38	0.00	0.25	0.25	0.00
CDI	0.57	0.22	−0.04	−0.04	−0.77	−0.38	0.00	−0.80	−0.80	0.00
SCARED	−1.82	−1.11	−1.08	−1.08	1.55	−0.38	0.00	0.02	0.02	0.00

EM, episodic migraine, CM, chronic migraine, CDI, Children Depression Inventory, SCARED, The Screen for Child Anxiety-Related Emotional Disorders.

**Figure 3:**
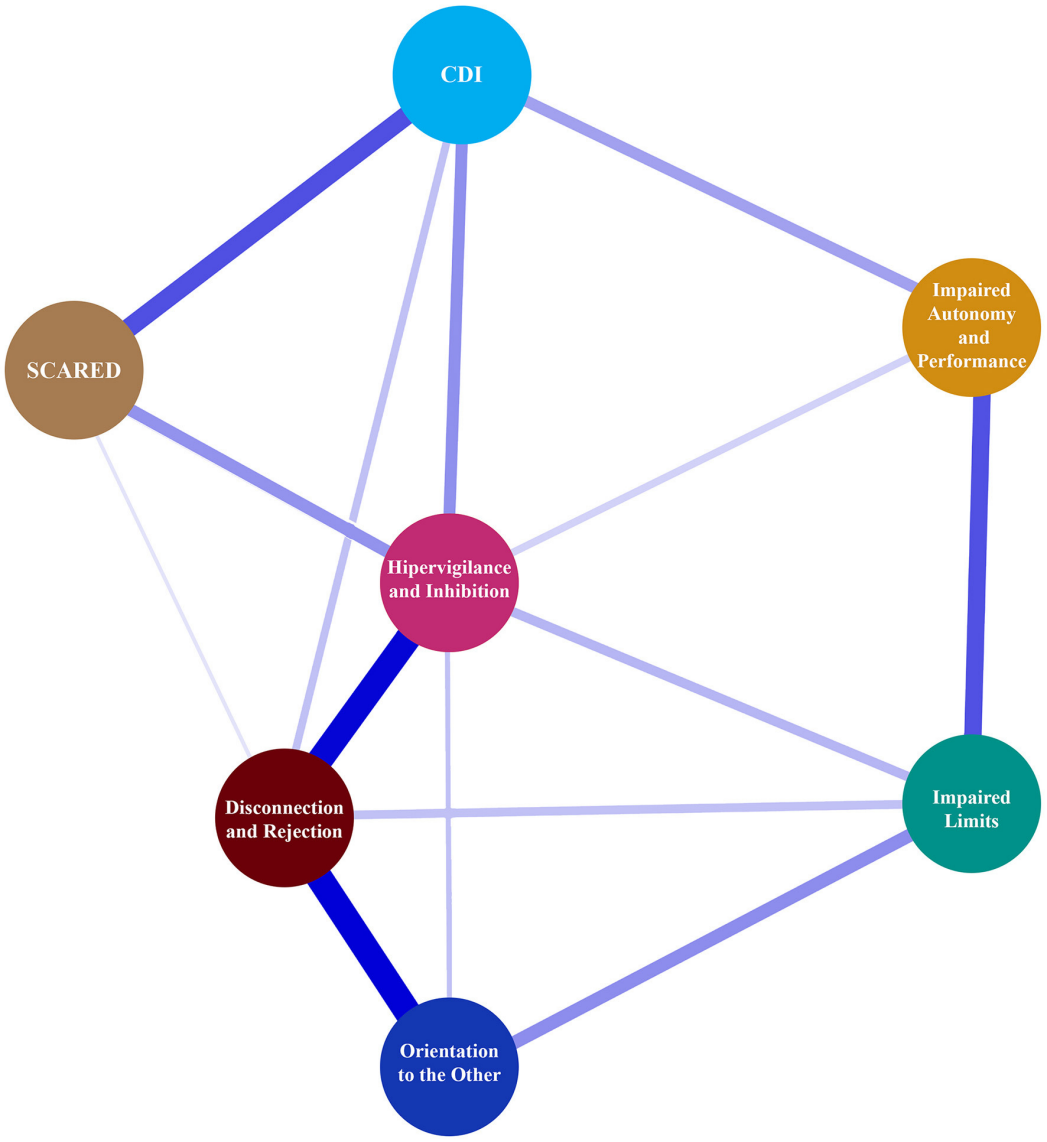
Network plot for adolescents between CDI, SCARED, and EMS domain with episodic migraine.

**Figure 4:**
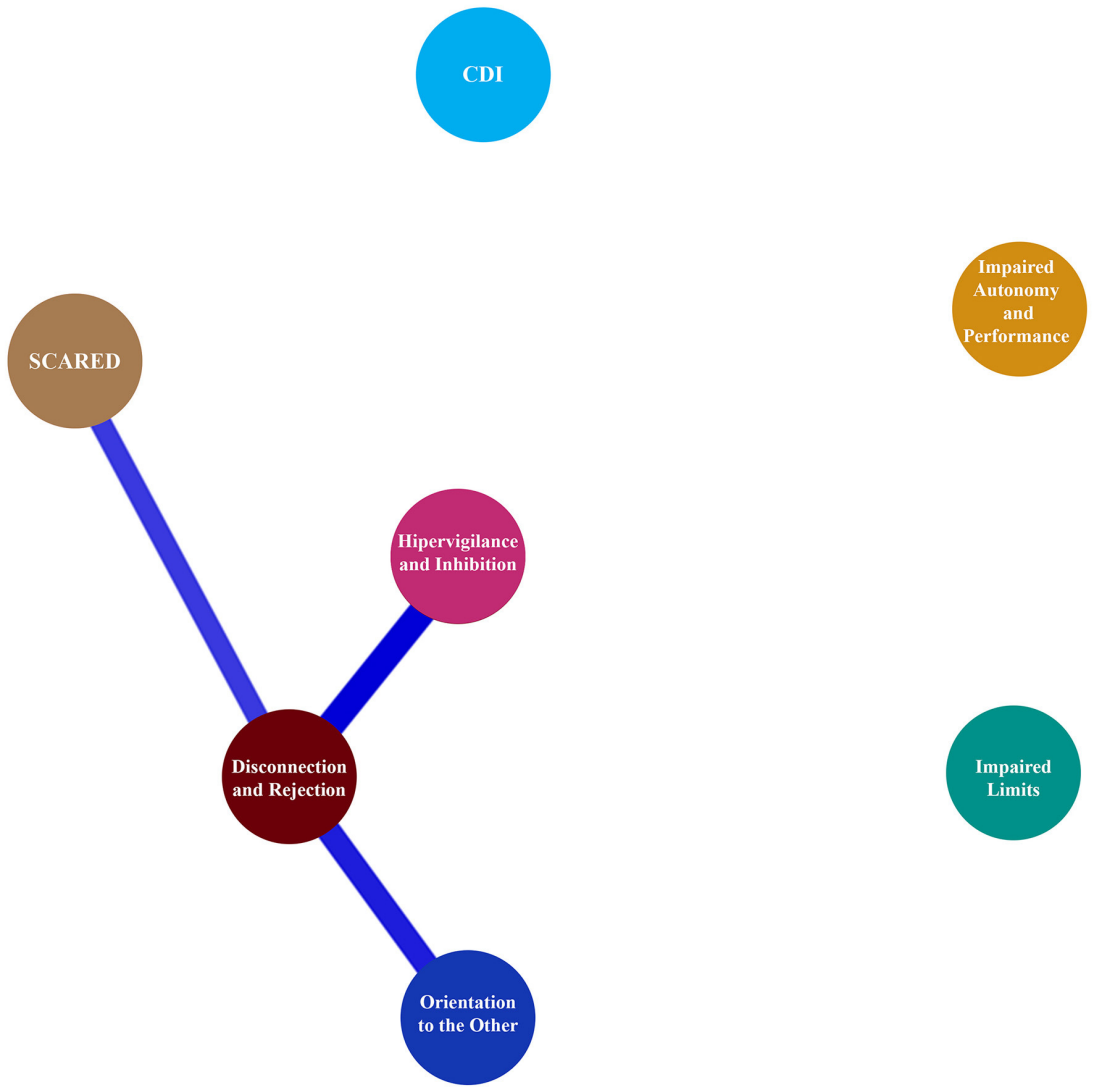
Network plot for adolescents between CDI, SCARED, and EMS domain with chronic migraine.

Network plots for adolescents with episodic (EM) and chronic (CM) migraine are illustrated in Figures 3, 4, respectively.

## 4. Discussion

This study investigated headache characteristics, EMSs, depression, anxiety, and the interrelationship of schema domains and others in adolescents with either EM or CM. The effects of age, current psychopathology, history of sexual abuse, anxiety, and depression on EMSs were also evaluated. Age, gender, sociodemographic characteristics, most pain features, and the core clinical characteristics of migraine did not differ significantly between groups. In bivariate comparisons, disconnection/rejection, other orientation schema domains, defectiveness/shame, mistrust/abuse, abandonment/instability, enmeshment of the undeveloped self, self-sacrifice, and subjugation schemas were more frequent in CM. Discriminant analysis revealed that the other orientation schema domain could correctly classify 99.3% of youths with EM and 96.3% of those with CM, for an overall accuracy of 83.8%. SCARED scores correctly classified 100.0% of youths with EM and none of those with CM, for an overall accuracy of 83.8%. Respondents with CM had higher depression and anxiety scores. In addition, the inclusion of sexual abuse and psychopathology as covariates did not affect the difference between the groups in terms of depressive and anxiety symptoms. Age, anxiety, and depression correlated with most of the EMS domains in adolescents with EM, and the relationship between anxiety, disconnection/rejection, and other orientation schema domains became evident with the migraine chronification.

Dogaheh et al. reported that EMSs and maladaptive coping styles (avoidance and overcompensation), which are used to overcome EMSs but have perpetuating long-term effects, were more common in patients with migraine and tension-type headaches than in a non-clinical group (10, 22). Bashiri Nejadian et al. revealed that EMSs may serve as a basis for emotional and behavioral tendencies and defensive coping styles in patients with migraine (23). Enayatian et al. also found that EMSs and clinical symptoms of migraine were mediated by dysfunctional attitudes (24). There seems to be an under-recognized, multidirectional relationship between EMSs, coping styles, attitudes, psychopathology, and migraine. Although there is an increasing interest in EMSs, the literature is still lacking in detailed studies about the possible role of EMSs in headaches, especially for children and adolescents. To our knowledge, only one study has been conducted to date on EMSs in adults with CM. It has been suggested that EMSs may explain a significant proportion of pain and that schema therapy may be effective in reducing the frequency and severity of headaches in CM (8). We conducted a study evaluating EMSs in adolescents with migraine and found that different EMSs between the sexes may play a role in migraine. It was the first study of EMSs in pediatric migraine (25). This study is also the first to investigate EMSs in episodic and chronic migraine in the pediatric age group.

CM is often transformed from EM, but the majority of people with EM do not develop CM. Many studies have evaluated risk factors to determine who may develop CM. Consistent results point to obesity, high attack frequency, medication overuse, snoring, and stressful life events (2, 4). Depression and anxiety are associated with a high risk of new-onset CM (2). Although there are many studies on stressful life events, depression, anxiety, and other psychiatric comorbidities in migraine, schemas, which are the basic structures of cognition, are understudied in both pediatric and adult migraine. This is the first study of EMSs in CM in adolescents, and the results show that there are different active schemas and schema domains in CM. In the study, sociodemographic/family characteristics, pain features, and the median number of psychopathologies were similar between the groups. Although causal explanations are not possible due to the design of the research, the difference in terms of EMSs in CM between these homogeneous groups is remarkable and merits further investigation into the underlying pathophysiology of CM.

Several studies confirm that psychiatric disorders are frequently seen in migraineurs (2). Mood and anxiety disorders are more commonly reported in CM (2). In our study, more than half of the cases in both groups were accompanied by a psychiatric disorder, showing the importance of psychopathology in migraine at an early age. Although there was no difference in the ratio of psychopathology between the groups, the fact that depressive and anxiety symptoms were high in CM indicates that the foundations of this course were laid at a younger age. Personality traits, childhood maltreatment, attachment, and emotional and behavioral problems are the most studied psychiatric topics in adult migraine (2, 9, 26), but further investigation on pediatric migraine may shed light on pathophysiology and chronification. Multi-center and community-based studies can elucidate the clinical features and accompanying psychopathologies of deeper cognitive structures called schemas in EM and CM, especially in children and adolescents.

Pain characteristics and core clinical features of migraine were not different between the groups. We also examined controversial clinical features, and two of them, lacrimation and photopsia, were more frequent in CM. Few studies have reported that cranial autonomic symptoms (CAS), which are the hallmarks of trigeminal autonomic cephalalgias, may occur in migraine (27). CAS is like a rule in pediatric migraine with a rate as high as 70% based on the ICHD-3 (28). Only one study reported that migraineurs with CAS had longer illness duration and longer headache episodes (29). Another study showed that CAS was related to the higher frequency of migraine attacks in children (30). To date, we have not found any data showing that lacrimation and photopsia are more common in CM. There is a need to investigate the extent of CAS in pediatric CM and its effects on chronicity. According to the results, EMSs associated with CAS may be independent risk factors for progression to treatment-resistant migraine. In early childhood, EMSs may lead to malformed neural networks, and their consequences in the long term may negatively affect mental health and headache courses.

Psychological stress influences the onset, severity, and course of many diseases. Psychological stress and EMSs have been studied in many diseases such as diabetes mellitus, psoriasis, atopic eczema, and irritable bowel syndrome (31–33). Migraine is a stress-related disorder. In this study, the presence of psychiatric disorders did not affect EMS scores, but sexual abuse did, especially in the domains of disconnection/rejection, impaired autonomy, and hypervigilance. The presence of sexual abuse did not affect self-reported depressive and anxiety symptoms, but psychopathologies did. These results suggest that EMSs may not be directly correlated with psychiatric disorder, but that EMSs may be important background factors in EM and CM, independent of psychopathologies. This hypothesis should be addressed with comprehensive longitudinal studies.

A recent review discovered evidence of a link between EMS and adolescent psychopathology. Depression, anxiety, eating pathology, borderline symptomatology, and externalizing behaviors were distinguished by EMSs (14). However, to date, there has been no study of EMSs in young patients with headaches. Tavallaii et al. revealed that divergent schemas were associated with headache pain severity, disability, and affective stress. They stated that the hypervigilance/inhibition domain had a predictive role in CM (8). Later, Ribas et al. confirmed the association between hypervigilance/inhibition and migraine, especially in female patients (34). Another study found that migraine and tension-type headaches were linked to mistrust/abuse, and self-sacrifice schemas in Iranian adults (10). In the current study, which supports previous adult studies, we found that defectiveness/shame, mistrust/abuse, abandonment/instability, enmeshment/undeveloped self, self-sacrifice and subjugation schemas, disconnection/rejection, and other orientations for schema domains were higher in adolescents with CM.

Recently, studies in adults have focused on the relationship between emotional problems, EMS, and migraine. Onen et al. speculated that the self-sacrifice schema may be associated with both depression and migraine (35). Previous studies reported that defensive styles/dysfunctional attitudes were linked to EMSs, emotional and behavioral symptoms, and migraine symptoms (14, 23, 24). According to Dogaheh, a set of EMSs could correctly predict 61% of the overall change in position in tension headaches or migraine groups in adults (10). In our study, the other orientation schema domain could correctly classify 99.3% of youths as EM and 96.3% as CM, for an overall accuracy of 83.8%. SCARED scores correctly classified 100.0% of adolescents with EM and none of those with CM, for an overall accuracy of 83.8%. Network analysis showed that CDI and SCARED scores had a moderately positive correlation with all EMS domains in youths with EM, and these correlations became evident between SCARED scores and disconnection/rejection and other orientation domains in youths with CM. This is important to consider in terms of which features may be unique to CM; further studies may answer this question.

This study has several limitations, including the limited number of patients, especially in the CM group, due to the retrospective design. The single-center, clinic-based, cross-sectional design limits the generalizability of the results. A larger study with a control group and follow-up studies are needed to confirm our findings. EMSs were evaluated using self-report scales, which may lead to reporting and recall bias. We did not use psychometric instruments to assess childhood adversity; instead, we relied on the reports of sexual abuse from respondents and their parents. The strength of this study is that we analyzed the results by taking into account comorbid psychopathology and abuse history. Another strength is that it is the first study to investigate EMSs, emotional factors, and their interrelationships within adolescents with EM and CM.

## Data availability statement

The raw data supporting the conclusions of this article will be made available by the authors, without undue reservation.

## Ethics statement

The studies involving human participants were reviewed and approved by Faculty of Medicine at Mersin University (Number: E-78017789-050.01.04-1659432 Date: 26/05/2021). Written informed consent from the participants' legal guardian/next of kin was not required to participate in this study in accordance with the national legislation and the institutional requirements.

## Author contributions

GG, OK, FT, and AÖzg designed the study and involved in data collection. MK, AT, DS, and BT analyzed the data. GG, AÖzd, OK, and AT wrote the original draft of the manuscript. AT, MK, FT, BT, DS, AÖzd, and AÖzg provided a critical review of the original draft of the manuscript. All authors read and approved the content of the manuscript.
